# Migraine and sleep apnea in the general population

**DOI:** 10.1007/s10194-010-0268-2

**Published:** 2010-12-17

**Authors:** Håvard Anton Kristiansen, Kari Jorunn Kværner, Harriet Akre, Britt Øverland, Michael Bjørn Russell

**Affiliations:** 1 Head and Neck Research Group, Research Centre, Akershus University Hospital, Postbox 65, 1478 Lørenskog, Norway; 2 Faculty Division Akershus University Hospital, University of Oslo, Oslo, Norway; 3 Department of Research and Education, Oslo University Hospital, Oslo, Norway; 4 Institute of Health Management and Health Economics, University of Oslo, Oslo, Norway; 5 Sleep Unit, Department of Otorhinolaryngology, Lovisenberg Diakonale Hospital, Oslo, Norway

**Keywords:** Migraine, Migraine without aura, Migraine with aura, Obstructive sleep apnea, Polysomnography and epidemiology

## Abstract

Objective is to investigate the relationship between migraine and obstructive sleep apnea in the general population. A cross-sectional population-based study. A random age and gender stratified sample of 40,000 persons aged 20–80 years residing in Akershus, Hedmark or Oppland County, Norway, were drawn by the National Population Register. A postal questionnaire containing the Berlin Questionnaire was used to classify respondents to be of either high or low risk of obstructive sleep apnea. 376 persons with high risk and 157 persons with low risk of sleep apnea aged 30–65 years were included for further investigations. They underwent an extensive clinical interview, a physical and a neurological examination by physicians, and in-hospital polysomnography. Those with apnea hypopnoea index (AHI) ≥5 were classified with obstructive sleep apnea. Migraine without aura (MO) and migraine with aura (MA) was diagnosed according to the International Classification of Headache Disorders. MO and MA occurred in 12.5 and 6.8% of the participants with obstructive sleep apnea. The logistic regression analyses showed no relationship between the two types of migraine and obstructive sleep apnea, with adjusted odds ratios for MO 1.15 (0.65–2.06) and MA 1.15 (0.95–2.39). Further, estimates using cutoff of moderate (AHI ≥ 15) and severe (AHI ≥ 30) obstructive sleep apnea, did not reveal any significant relationship between migraine and the AHI. Migraine and obstructive sleep apnea are unrelated in the general population.

## Introduction

A relationship between headache and sleep has been recognized for a long time [1]. Migraine attacks may be precipitated by sleep deprivation and patients with migraine often experience relief of their pain with sleep, while rest without sleep has been shown to be less effective [2, 3]. A recent study showed a significant association between primary headaches including migraine and severe sleep disturbances measured by two validated sleep questionnaires [4]. One of the most common sleep disorders is obstructive sleep apnea syndrome, with an estimated prevalence of 2–4% among middle-aged adults [5, 6].

Obstructive sleep apnea syndrome is defined as at least five apneas or hypopneas per hour of sleep in conjunction with symptoms such as excessive daytime sleepiness. When obstructive sleep apnea is defined solely by an apnea hypopnea index (AHI) of ≥5, the estimated prevalence among middle-aged adults is approximately 20% in the general population [5, 7, 8]. This is a disorder with partial or complete obstruction of the upper airways during sleep which constitutes hypopnea and apnea and will typically result in repeated airflow cessation, oxygen desaturation and sleep disruption. The disruption of sleep may then result in one or more of the following: excessive daytime sleepiness, unrefreshing sleep, daytime fatigue or reduced cognitive function [9].

Sleep apnea headache is recognized in the in the International Classification of Headache Disorders (ICHD II) as a brief recurrent morning headache in the presence of an apnea hypopnea index (AHI) of ≥5 [10]. There is however, still controversy regarding the association between primary headaches and obstructive sleep apnea. The apnea-related headache may present itself as migraine, tension-type, cluster or a non-specific headache, and several studies have found it to merely be a non-specific symptom with no clear relationship with obstructive sleep apnea [11–14].

The aim of the present study was to investigate the relationship between migraine without aura (MO) and migraine with aura (MA), and obstructive sleep apnea in the general population.

## Methods

### Sampling and representativeness

**Fig. 1:**
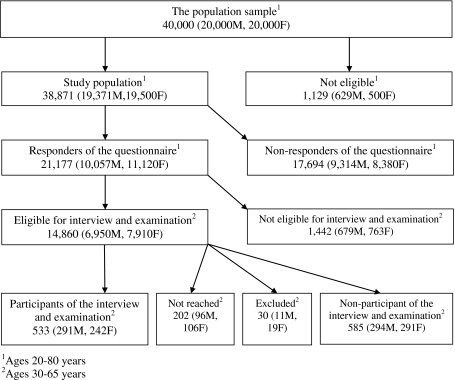
Flow chart of the study population according to type of participation, *M* and *F* denotes males and females

This is a cross-sectional population-based study. An age and gender stratified random sample of 40,000 persons aged 20–80 years were drawn by the National Population Register. Each of the ages 30, 35, 40, 45, 50, 55 and 60 years included 2,000 persons of each gender, while the ages 20, 25, 65, 70, 75 and 80 years included 1,000 persons of each gender. The participants were residing in Akershus, Hedmark or Oppland County, Norway. The counties have both rural and urban areas, and Akershus County is situated in close proximity to Oslo. Data from Statistics Norway has shown that the sampling area was representative for the total Norwegian population regarding age, gender, marital status and level of education [15]. The employment rate was equal, but employment in trade, hotel/restaurant and transport were overrepresented, while industry, oil and gas and financial services were underrepresented in the sampling area as compared to the total Norwegian population. As shown in Fig. 1, the sample size was reduced to 38,871 because of error in the address list (*n* = 1,024), multihandicap (*n* = 4), dementia (*n* = 23), insufficient Norwegian language skills (*n* = 3) and deceased (*n* = 75). All participants received a mailed standard letter containing information about the project and a short questionnaire including the Berlin Questionnaire. The Berlin Questionnaire was used to classify respondents to be of either high or low risk of obstructive sleep apnea [16]. If the questionnaire evoked no response, a second mail was issued. The replies could either be on paper or electronically. The overall response rate was 54.5% (21,177/38,871), and it was significantly higher among women than men (*n* = 11,120 vs. *n* = 10,057; *p* < 0.001). A total of 1,442 questionnaires were not eligible. This was due to late response (*n* = 41), not containing a telephone number necessary for re-contact (*n* = 729) and incompletely filled in questionnaires that could not be classified as high or low risk for obstructive sleep apnea (*n* = 672). An age and gender stratified sample of the respondents aged 30–65 years were then invited by mail to a clinical evaluation and contacted by telephone. The clinical evaluation was conducted over a period of 2 years. If they could not be reached within three attempts, no further attempts were made (*n* = 202). Other exclusion criteria were: use of continuous positive airway pressure (*n* = 10), pregnancy (*n* = 9), lack of Norwegian language skills (*n* = 5) and severe physical impairment (*n* = 4). A total of 378 persons with high risk and 157 persons with low risk of sleep apnea were included for further investigations. In case of technical failure in the polysomnography (PSG) recordings, the participants were asked to return for a second recording. Since two persons refrained from such a second polysomnography recording, the final study sample in the present study comprised of 533 (376 high risk and 157 low risk) persons. 585 persons refrained from participating; yielding a participation rate of the interview and examination of 47.7%.

### Clinical evaluation

The participants were all admitted to Akershus University Hospital (Stensby Hospital), Norway, and underwent an extensive clinical interview including a semi-structured headache interview and a physical and a neurological examination by one of three physicians. The physicians were blinded regarding the participants replies on the questionnaire. The International Classification of Headache Disorders (ICHD II) was applied [10]. The Hospital Anxiety and Depression Scale (HADS) was used to screen for depression [17]. The replies were dichotomized and depression was defined by a score of ≥8 on the subscale of depression (HADS-D) [18]. Excessive daytime sleepiness was assessed by the Epworth Sleepiness Scale [19]. The results were dichotomized into scores ≤10 and >10, the latter is considered to represent clinically significant excessive daytime sleepiness [20]. Body mass index (kg/m^2^) was calculated from measured weight and height. All participants then underwent in-hospital polysomnography (PSG) performed on standard, multichannel, Embla^TM^, PSG devices (ResMed Corp Poway, CA, USA). The recordings included a two-channel electroencephalograph (C4/A1, C3/A2 according to the 10-20 international electrode placement system), a two-channel electrooculogram, a one-channel submental electromyogram, leg EMG (tibialis), SaO_2_, breathing movements (Respitrace; Ambulatory Monitoring, Ardsley, NY, USA), air flow (Pro-Tech, Woodinville, WA, USA) and body position. All electrophysiological signals were pre-amplified, stored and subsequently scored (30-s epochs using Somnologica 3.2 software package, Flaga-Medcare, Buffalo, NY, USA) according to the Rechtshaffen and Kales scoring manual by two US board certified PSG technicians who were blinded to the result of the Berlin Questionnaire [21]. Arousals were documented and classified [22]. Obstructive apneas were scored when at least a 90% decrease of flow occurred for more than 10 s. Hypopneas were defined as a 30% decrease in flow for more than 10 s with subsequent oxygen desaturation of at least 4%. The apnea hypopnoea index (AHI) was calculated as the average of total number of apneas and hypopneas per hour of sleep. In this study the participants with apnea hypopnoea index (AHI) ≥5 were classified with obstructive sleep apnea.

### Statistical analyses

Statistical analyses were performed using SPSS Base System for Windows 16.0. Chi-square tests and logistic regression modeling with 5% level of significance were used. Fisher’s exact test was used when appropriate. The Student’s *t* test and Mann–Whitney *U* test were used in comparing normally and non-normally distributed continuous variables. In our multivariate logistic regression model obstructive sleep apnea was used as the dependent variable, while migraine, depression, gender, body mass index and age were independent variables.

### Ethical issues

The project was approved by The Regional Committees for Medical Research Ethics and the Norwegian Social Science Data Services.

## Results

Participants and non-participants were not significantly different regarding self-reported migraine (31.0 vs. 28.8%, *p* = 0.43), depression (10.1 vs. 11.9%, *p* = 0.34), simple snoring (89.8 vs. 87.2%, *p* = 0.37), gender (male, 54.3 vs. 50.1%; *p* = 0.16) or age (mean age 48.6 vs. 48.8 years, *p* = 0.75), while simple snoring was overrepresented in the low-risk group, as compared to all low-risk respondents of the questionnaire (82.2 vs. 45.1%, *p* < 0.001).

The distribution of demographic and clinical characteristics of the sample is shown in Table 1. Respondents with high risk of obstructive sleep apnea according to the Berlin Questionnaire were oversampled, resulting in obstructive sleep apnea occurring in 55.5% (296/533) of the participants. The mean body mass index (kg/m^2^) in the study sample was 28.9 (SD 5.0). MO was diagnosed in 12.5% (37/296) and 14.3% (34/237) of the participants with and without obstructive sleep apnea. When using cutoff of moderate (AHI ≥ 15) and severe (AHI ≥ 30) obstructive sleep apnea, the prevalence of MO was 12.2% (20/164) and 8.4% (7/83), respectively. MA was diagnosed in 6.8% (20/296) and 7.6% (18/237) of the participants with and without obstructive sleep apnea. When using cutoff of moderate (AHI ≥ 15) and severe (AHI ≥ 30) obstructive sleep apnea, the prevalence of MA was 4.9% (8/164) and 7.2% (6/83), respectively.

**Table 1:** Demographic and clinical characteristics of the study sample

	Male (*N* = 291)		Female (*N* = 242)		All (*N* = 533)	
	%	*n*	%	*n*	%	*n*
Age (years)^a^						
30 and 35	20.6	60	22.3	54	21.4	114
40 and 45	24.4	71	26.0	63	25.1	134
50 and 55	25.4	74	25.6	62	25.5	136
60 and 65	29.6	86	26.0	63	28.0	149
Depression						
Yes	8.4	24	12.4	30	10.2	54
No	91.6	263	87.6	212	89.8	475
Body mass index (kg/m^2^)						
<18.5	0	0	0.4	1	0.2	1
18.5–24.9	17.5	51	29.3	71	22.9	122
25.0–29.9	38.8	113	35.1	85	37.1	198
≥30.0	43.6	127	35.1	85	39.8	212
Excessive daytime sleepiness						
Yes	29.7	86	35.3	85	32.2	171
No	70.3	204	64.7	156	67.8	360
Obstructive sleep apnea (AHI ≥ 5)						
Yes	65.6	191	43.4	105	55.5	296
No	34.4	100	56.6	137	44.5	237
AHI ≥ 15						
Yes	40.5	118	19.0	46	30.8	164
No	59.5	173	81.0	196	69.2	369
AHI ≥ 30						
Yes	23.4	68	6.2	15	15.6	83
No	76.6	223	93.8	227	84.4	450
Migraine without aura, *n* (%)						
Yes	8.2	24	19.4	47	13.3	71
No	91.8	267	80.6	195	86.7	462
Migraine with aura, *n* (%)						
Yes	4.1	12	10.7	26	7.1	38
No	95.9	279	89.3	216	92.9	495

^a^Age at sampling of questionnaire data

Table 2 illustrates the clinical characteristics of MO and MA. One female participant with MA had exclusively sensory, motor and speech disturbances.

**Table 2:** Clinical characteristics of migraine without aura and migraine with aura

	Migraine without aura (*N* = 71)		Migraine with aura (*N* = 38)	
	%	*n*	%	*n*
Lifetime number of attacks				
2–4	3	2^a^	5	2
5–9	3	2	3	1
10–49	21	14	43	16
50–99	19	13	11	4
≥100	54	37	38	14
Frequency (days per month)				
<1	58	40	58	22
1–6	38	26	40	15
7–14	3	2	3	1
≥15	1	1	0	0
Duration (h)				
<4	17	12^a^	32	12
4–24	65	45	40	15
>24	17	12	29	11
Pain characteristics				
Unilateral location	79	55	43	16
Pulsating quality	89	62	70	26
Moderate/severe intensity	99	69	97	37
Daily activity inhibited	99	69	95	36
Accompanying symptoms				
Nausea	80	56	79	30
Photophobia	91	64	92	35
Phonophobia	77	54	74	28
Vomiting	53	37	63	24
Migraine aura				
Visual disturbances	–	–	97	37
Sensory disturbances	–	–	24	9
Motor disturbances	–	–	11	4
Speech disturbances	–	–	8	3
Migraine age at onset, mean (SD)	24.5	11.2	24.9	8.7

^a^Probable migraine without aura

The polysomnographic characteristics are shown in Table 3. Due to the high number of participants with obstructive sleep apnea, the number of minutes with deep sleep (S3 and S4) and REM sleep were somewhat low and the mean sleep efficiency was below 85%. Comparing these normally and non-normally distributed continuous variables shown in Table 3 did not reveal any significant differences between participants with MO, MA and those without migraine.

**Table 3:** Polysomnographic characteristics of the study sample

	Migraine without aura (*N* = 71)		Migraine with aura (*N* = 38)		No migraine (*N* = 431)	
	Mean	SD	Mean	SD	Mean	SD
Total sleep time (min)	414.0	92.0	418.4	88.9	409.6	82.9
Sleep efficiency (%)	84.1	10.6	83.0	13.2	83.1	11.6
S1 of total sleep time (%)	6.7	6.8	6.5	5.9	6.8	6.9
S2 of total sleep time (%)	55.6	9.1	54.4	10.2	52.5	10.4
S3 of total sleep time (%)	5.8	2.7	5.8	3.4	6.7	3.5
S4 of total sleep time (%)	15.4	5.9	16.7	6.2	16.5	7.5
REM of total sleep time (%)	16.8	6.4	17.1	7.2	18.1	6.2
Sleep latency (min)	66.9	115.4	47.2	79.4	40.9	78.0
REM latency from sleep onset (min)	124.8	78.1	125.5	83.0	117.9	67.9
Arousal index	17.8	17.1	18.3	19.9	17.4	13.5
Apnea hypopnoea index (AHI)	11.2	14.1	12.3	17.1	14.6	18.9
Oxygen desaturation index (ODI)	11.5	14.5	11.5	15.8	14.3	17.9
Average oxygen saturation (%)	94.6	1.6	94.8	1.5	94.4	1.9
Lowest oxygen saturation (%)	85.3	5.9	85.3	6.0	84.6	6.5
Average desaturation (%)	5.1	1.0	5.3	1.6	5.3	1.5

Tables 4, 5 shows the odds ratios for obstructive sleep apnea by MO, depression, gender, body mass index and age. Neither the crude nor the adjusted probability for MO was significantly increased among participants with obstructive sleep apnea. A similar non-significant result is shown for the logistic regression model regarding MA. Finally, to minimize the risk of a type-2 error, we combined the data of MO and MA in a similar logistic regression model as shown in Tables 4, 5. In this analysis the adjusted odds ratio for migraine in participants with obstructive sleep apnea was 1.31 (0.79–2.17). We did however, find a statistically significant relationship between excessive daytime sleepiness and migraine with a crude odds ratio of 1.94 (1.24–3.02) and an adjusted odds ratio of 1.66 (1.04–2.65).

**Table 4:** Crude and adjusted odds ratios (cOR and aOR) with 95% confidence intervals (CI) for obstructive sleep apnea by migraine without aura, depression, gender, body mass index and age

	cOR	95% CI	aOR^a^	95% CI
Migraine without aura				
No	Ref		Ref	
Yes	0.85	0.52–1.41	1.15	0.65–2.06
Depression				
No	Ref		Ref	
Yes	0.93	0.53–1.63	0.95	0.49–1.83
Gender				
Female	Ref		Ref	
Male	2.49	1.76–3.54	2.62	1.76–3.89
Body mass index	1.13	1.08–1.17	1.14	1.09–1.20
Age	1.07	1.05–1.08	1.07	1.05–1.09

^a^Adjustments performed as a multiple logistic regression analysis. All variables are adjusted for the other independent variables in the table

**Table 5:** Crude and adjusted odds ratios (cOR and aOR) with 95% confidence intervals (CI) for obstructive sleep apnea by migraine with aura, depression, gender, body mass index and age

	cOR	95% CI	aOR^a^	95% CI
Migraine with aura				
No	Ref		Ref	
Yes	0.88	0.46–1.71	1.15	0.55–2.39
Depression				
No	Ref		Ref	
Yes	0.93	0.53–1.63	0.96	0.50–1.84
Gender				
Female	Ref		Ref	
Male	2.49	1.76–3.54	2.61	1.76–3.87
Body mass index	1.13	1.08–1.17	1.14	1.09–1.20
Age	1.07	1.05–1.08	1.07	1.05–1.09

^a^Adjustments performed as a multiple logistic regression analysis. All variables are adjusted for the other independent variables in the table

## Discussion

### Results

The main finding in this population-based cross-sectional study was the lack of relationship between MO, MA and all migraine, and obstructive sleep apnea. This is in concurrence with two previous clinical population studies from France and Norway [14, 23]. In the French study which recruited patients referred to a sleep laboratory because of snoring, the prevalence of migraine was higher among snorers (40.7%) than in patients with sleep apnea syndrome (28.3%), but the difference was not statistically significant (*p* = 0.18) [23]. The Norwegian study recruited patients referred to a neurologist because of headache. They also found a higher prevalence of migraine among patients without obstructive sleep apnea (40%) than among patients with obstructive sleep apnea (29%), but the result was not statistically significant (*p* = 0.39) [14].

The consistency of our results is further emphasized by the fact that mild (AHI ≥ 5), moderate (AHI ≥ 15) and severe (AHI ≥ 30) obstructive sleep apnea showed exactly the same. This lack of dose–response relationship between headache and severity of obstructive sleep apnea has previously been reported in two case–control studies from USA and Norway, respectively [12, 24].

In contrast, another Norwegian population-based survey found that severe sleep disturbances were five times more likely among migraineurs than in headache-free individuals [4]. Sleep disturbances in that survey was based on the Karolinska Sleep Questionnaire with a score in the upper quartile. This questionnaire assesses snoring, apnea, insomnia, daytime sleepiness and restless legs syndrome, and in the analysis of the separate items they did not find any differences in the prevalence of snoring or apnea between migraineurs and headache-free individuals. Their finding that excessive daytime sleepiness is more likely among migraineurs was confirmed in our study.

### Methodological considerations

The strengths of this study were the use of interview and examination by physicians regarding the diagnoses of MO and MA as well as the use of PSG in diagnosing obstructive sleep apnea in participants from the general population. Although the response rate to the questionnaire was relatively low, similar replies to the first and second issued questionnaire as well as the electronic responses suggest that responders and non-responders are not different. A previous Danish epidemiological survey found no significant difference in the frequency of migraine among responders and non-responders [25]. In addition, the response rate is comparable to that of other sleep-related epidemiologic studies [26, 27].

The relatively low participation rate may introduce a selection bias. However, participants and non-participants were not significantly different regarding self-reported migraine, depression, simple snoring, gender or age. Another possible selection bias is the fact that respondents with high risk of obstructive sleep apnea were oversampled. As expected, this resulted in a high prevalence of obstructive sleep apnea (55.5%) and excessive daytime sleepiness (32.2%) in our sample. However, we do not believe that increased sleepiness has influenced the estimated relationship between migraine and obstructive sleep apnea, since no significant associations were found. Regarding the difference between the participants and the study population, we discovered that self-reported simple snoring was overrepresented in the low-risk group in the study sample, as compared to all respondents of the questionnaire with low risk. Since there may be a relationship between snoring and headache, this may have introduced a misclassification bias which contributed to a slight overestimation of headache in participants without obstructive sleep apnea in the current study [28, 29]. This will not, however, influence our finding that migraine and the AHI was non-significantly related. Finally, it cannot be completely ruled out that the use of single in-patient PSG may be a potential limitation to our study [30]. Although the mean total sleep time in this sample was 411.7 min, which may represent a first night effect, we believe the latter is more important in measuring of the sleep quality than in diagnosing of obstructive sleep apnea.

## Conclusion

There seems to be no clear relationship between migraine without and with aura, and obstructive sleep apnea in the general population.

## References

[1] Dodick DW, Eross EJ, Parish JM, Silber M (2003). Clinical, anatomical, and physiologic relationship between sleep and headache. Headache.

[2] Inamorato E, Minatti-Hannuch SN, Zukerman E (1993). The role of sleep in migraine attacks. Arq Neuropsiquiatr.

[3] Wilkinson M, Williams K, Leyton M (1978). Observations on the treatment of an acute attack of migraine. Res Clin Stud Headache.

[4] Odegard SS, Engstrom M, Sand T, Stovner LJ, Zwart JA, Hagen K (2010). Associations between sleep disturbance and primary headaches: the third Nord-Trondelag Health Study. J Headache Pain.

[5] Young T, Palta M, Dempsey J, Skatrud J, Weber S, Badr S (1993). The occurrence of sleep-disordered breathing among middle-aged adults. N Engl J Med.

[6] Kripke DF, Ancoli-Israel S, Klauber MR, Wingard DL, Mason WJ, Mullaney DJ (1997). Prevalence of sleep-disordered breathing in ages 40–64 years: a population-based survey. Sleep.

[7] Duran J, Esnaola S, Rubio R, Iztueta A (2001). Obstructive sleep apnea-hypopnea and related clinical features in a population-based sample of subjects aged 30 to 70 yr. Am J Respir Crit Care Med.

[8] Bixler EO, Vgontzas AN, Lin HM, Ten HT, Rein J, Vela-Bueno A (2001). Prevalence of sleep-disordered breathing in women: effects of gender. Am J Respir Crit Care Med.

[9] Sleep-related breathing disorders in adults: recommendations for syndrome definition and measurement techniques in clinical research. The Report of an American Academy of Sleep Medicine Task Force (1999) Sleep 22:667–689 10450601

[10] Headache Classification Subcommittee of the International Headache Society (2004) The International Classification of Headache Disorders: 2nd edn. Cephalalgia 24 Suppl 1:9–160 10.1111/j.1468-2982.2003.00824.x 14979299

[11] Alberti A, Mazzotta G, Gallinella E, Sarchielli P (2005). Headache characteristics in obstructive sleep apnea syndrome and insomnia. Acta Neurol Scand.

[12] Aldrich MS, Chauncey JB (1990). Are morning headaches part of obstructive sleep apnea syndrome?. Arch Intern Med.

[13] Poceta JS, Dalessio DJ (1995). Identification and treatment of sleep apnea in patients with chronic headache. Headache.

[14] Jensen R, Olsborg C, Salvesen R, Torbergsen T, Bekkelund SI (2004). Is obstructive sleep apnea syndrome associated with headache?. Acta Neurol Scand.

[15] Russell MB, Kristiansen HA, Saltyte-Benth J, Kvaerner KJ (2008). A cross-sectional population-based survey of migraine and headache in 21, 177 Norwegians: the Akershus sleep apnea project. J Headache Pain.

[16] Netzer NC, Stoohs RA, Netzer CM, Clark K, Strohl KP (1999). Using the Berlin Questionnaire to identify patients at risk for the sleep apnea syndrome. Ann Intern Med.

[17] Zigmond AS, Snaith RP (1983). The hospital anxiety and depression scale. Acta Psychiatr Scand.

[18] Bjelland I, Dahl AA, Haug TT, Neckelmann D (2002). The validity of the Hospital Anxiety and Depression Scale. An updated literature review. J Psychosom Res.

[19] Johns MW (1991). A new method for measuring daytime sleepiness: the Epworth sleepiness scale. Sleep.

[20] Johns MW (1994). Sleepiness in different situations measured by the Epworth Sleepiness Scale. Sleep.

[21] Rechtschaffen A, Kales A (1968) A manual of standardized terminology, techniques and scoring system for sleep stages of human subjects. Los Angeles, University of California, Brain Information Service/Brain Research Institute (ref type: generic)

[22] Bonnet M, Carley D, Carskadon M, Easton P, Guilleminault C, Harper R (1992). EEG arousals: scoring rules and examples: a preliminary report from the Sleep Disorders Atlas Task Force of the American Sleep Disorders Association. Sleep.

[23] Neau JP, Paquereau J, Bailbe M, Meurice JC, Ingrand P, Gil R (2002). Relationship between sleep apnoea syndrome, snoring and headaches. Cephalalgia.

[24] Sand T, Hagen K, Schrader H (2003). Sleep apnoea and chronic headache. Cephalalgia.

[25] Russell MB, Rasmussen BK, Thorvaldsen P, Olesen J (1995). Prevalence and sex-ratio of the subtypes of migraine. Int J Epidemiol.

[26] Ancoli-Israel S, Roth T (1999) Characteristics of insomnia in the United States: results of the 1991 National Sleep Foundation Survey. I. Sleep 22(Suppl 2):S347–S353 10394606

[27] Pallesen S, Nordhus IH, Omvik S, Sivertsen B, Tell GS, Bjorvatn B (2007). Prevalence and risk factors of subjective sleepiness in the general adult population. Sleep.

[28] Jennum P, Hein HO, Suadicani P, Gyntelberg F (1994). Headache and cognitive dysfunctions in snorers. A cross-sectional study of 3323 men aged 54 to 74 years: the Copenhagen Male Study. Arch Neurol.

[29] Ulfberg J, Carter N, Talback M, Edling C (1996). Headache, snoring and sleep apnoea. J Neurol.

[30] Stepnowsky CJ, Orr WC, Davidson TM (2004). Nightly variability of sleep-disordered breathing measured over 3 nights. Otolaryngol Head Neck Surg.

